# Targeting Inflammatory Imbalance in Chronic Kidney Disease: Focus on Anti-Inflammatory and Resolution Mediators

**DOI:** 10.3390/ijms26073072

**Published:** 2025-03-27

**Authors:** Rosaria Margherita Rispoli, Ada Popolo, Vincenzo De Fabrizio, Roberta d’Emmanuele di Villa Bianca, Giuseppina Autore, Jesmond Dalli, Stefania Marzocco

**Affiliations:** 1 Department of Pharmacy, University of Salerno, Via Giovanni Paolo II 132, 84084 Fisciano, Italy; r.rispoli20@studenti.unisa.it (R.M.R.); apopolo@unisa.it (A.P.); defabriziov@gmail.com (V.D.F.); autore@unisa.it (G.A.); 2 PhD Program in Drug Discovery and Development, University of Salerno, Via Giovanni Paolo II 132, 84084 Fisciano, Italy; 3 Department of Pharmacy, University of Naples Federico II, 80131 Naples, Italy; demmanue@unina.it; 4 William Harvey Research Institute, Barts and The London School of Medicine and Dentistry, Queen Mary University of London, Charterhouse Square, London E1 4NS, UK; j.dalli@qmul.ac.uk; 5 Centre of Inflammation and Therapeutic Innovation, Queen Mary University of London, London E1 4NS, UK

**Keywords:** chronic kidney disease, chronic inflammation, inflammatory markers, pro-resolving mediators

## Abstract

Chronic kidney disease (CKD) is a condition caused by the gradual decline of renal function that approximatively affects 10–12% of the world population, thus representing a public health priority. In CKD patients, chronic and systemic low-grade inflammation is observed, and it significantly contributes to disease development and progression, especially for patients with advanced disease. It also results in CKD-associated complications and increased mortality. The low-grade inflammation is due to different factors, such as the decline of glomerular filtration rate, increased immune system activation, reactive oxygen species release, and intestinal homeostasis. Therefore, the possibility to control chronic low-grade inflammation in CKD deserves great attention. In this review, we will examine the current possible pharmacological approaches to counteract the inflammatory state in CKD, focusing our attention both on the pro-inflammatory factors and the pro-resolving mediators involved in CKD inflammatory state.

## 1. Introduction

Chronic kidney disease (CKD) is a complex and multifactorial pathological condition that affects more than 800 million individuals in the world and represents a global health problem, becoming a leading cause of death worldwide with an increase in deaths over the past two decades [1,2,3]. The main causes of CKD are hypertension, diabetes, immune-mediated diseases, glomerulonephritis, tubulointerstitial disease, and inherited kidney diseases. CKD treatment aims to slow down the progression of kidney damage, primarily by managing the underlying cause. However, even with proper management of the underlying cause, kidney damage may continue to progress, leading to end-stage renal disease (ESRD), which is life-threatening without artificial filtration through dialysis or a kidney transplant. As renal replacement therapy only partially corrects the uremic state, kidney transplantation is the treatment of choice, although it requires lifelong immunosuppression [4]. The number of people receiving renal replacement therapy, which aims to replace nonendocrine kidney function in patients with renal failure using techniques such as intermittent hemodialysis, continuous hemofiltration and hemodialysis, and peritoneal dialysis, exceeds 2.5 million and is expected to double to 5.4 million by 2030 [5]. Dialysis is the most common treatment for CKD. It contributes to reduce the accumulation of high levels of metabolic end products, normally excreted by a healthy kidney, uremic toxins, the accumulation of which is clinically relevant in CKD progression and related complications [6,7,8,9,10,11]. In fact, CKD affects more than just the kidneys, as patients frequently experience various complications, including hypertension, cardiovascular diseases, anemia, metabolic acidosis [12], impaired immune response, mineral and bone disorders, and neurological complications [13].

Among these complications, cardiovascular dysfunction and infections, exacerbated by an impaired immune response, have been identified as major contributors to increased morbidity and mortality [14]. Inflammation also plays a central role in CKD-related complications [15]. While inflammation is essential for defending against infections, its dysregulation can trigger harmful effects, including the excessive production of pro-inflammatory cytokines, which sustain a persistent inflammatory response throughout the body [13]. Patients with CKD suffer from chronic and low-grade inflammation. This worsens with the degree of renal failure [11]. Over the past two decades, numerous lines of evidence point to chronic inflammation as a pivotal element of the uremic phenotype, which significantly contributes to the progression and acceleration of CKD-related complications. Moreover, in addition to the immune system’s activation, pathogens and other abnormal metabolites (e.g., uremic toxins) can trigger chronic inflammation, thus contributing to the CKD-associated inflammatory state [16,17], Despite great progress in understanding chronic inflammation, how to prevent and treat it, mostly in association with other chronic diseases, such as CKD, remains a challenging problem.

## 2. Materials and Methods

To identify relevant studies, an accurate literature search was performed using PubMed (MEDLINE), Scopus, and Web of Science databases. These platforms were searched using keywords such as “chronic kidney disease,” “CKD”, “inflammation in CKD”, “pro-resolving mediators in CKD”, “pro-inflammatory factors in CKD”, and “oxidative stress in CKD.” Data from relevant studies were selected based on factors such as relevance of the publication, research methodology, results, statistical significance, and publication date, up to 18 March 2025. In addition, a manual search of the reference lists of key studies was conducted to identify additional relevant information. A total of 204 publications were selected, including systematic reviews, research articles, and meta-analyses.

## 3. Inflammation in CKD

Inflammation is a key regulator of host defense and plays a pivotal role in tissue repair, regeneration, and maintenance of homeostasis. Several highly conserved pathways govern the initiation and progression of the inflammatory response, ensuring an effective yet controlled reaction to eliminate pathogens and remove debris from damaged tissues, thereby promoting tissue repair. However, if the process is chronic and poorly controlled, it can promote a wide range of complications. Chronic and unresolved inflammation could result in scarring, dysfunction, and organ failure. An inducing stimulus, such as tissue injury or a body, triggers the inflammatory cascade, inducing pro-inflammatory cytokines cascade, with a consequent blood flow increase, upregulation of chemical mediators, and leukocyte infiltration [18]. The discontinuation of this response is then mediated by anti-inflammatory molecules. However, in the presence of discontinuation or endurance of pro-inflammatory mediators, low-grade inflammation may persist. Several factors can contribute to the persistence of a low-grade inflammatory state, including poor diet and nutrition, gut microbiota, childhood infection, and stress [19].

Persistent and unresolved inflammation is also a typical characteristic of numerous chronic conditions, where the inflammatory response is induced by internal triggers (e.g., atherosclerosis, kidney disease, diabetes, metabolic syndrome, non-alcoholic fatty liver disease, and cognitive decline) [3].

Chronic inflammation is one of the most important features of CKD, which is mostly advanced and increasingly associated with deterioration of kidney function. Evidence suggests that C-reactive protein (CRP) is elevated in more than half of CKD patients from stage 3 onwards, with a higher incidence in patients with ESRD [20]. However, the exact timing between the onset of inflammation and CKD remains unclear. It should be noted that the kidney is at higher risk of inflammation-related damage than many other organs [21], as it is one of the most metabolically active organs and is sensitive to many damaging factors, such as oxidative damage and alteration in calcium-phosphorus metabolism and bone disease, hyperhomocysteinemia, and malnutrition with uremic sarcopenia [22]. The kidneys typically receive 25% of the total blood volume but lack the anti-inflammatory defense found in other highly vascularized organs, such as antioxidants or detoxifying agents present in the liver. Additionally, alterations in intrarenal microcirculatory regulation caused by chronic inflammation lead to kidney damage, accelerating the progression of kidney disease. Many pro-inflammatory cytokines, chemokines, and fibrosis mediators are present in the renal tubules, which play a critical role in responding to kidney insults and injuries, and these mediators are tightly regulated. The causes of chronic inflammation in chronic kidney disease (CKD) are multifactorial, influenced by several factors that arise during the disease. These include pro-inflammatory cytokine production, oxidative stress, dialysis, intestinal dysbiosis, uremic toxin retention, and altered adipose tissue metabolism, all of which contribute to inflammation in CKD [22,23,24].

Progressive and chronic renal dysfunction is characterised by an increase in oxidative stress, which can be considered a consequence of the inefficient role of the renal antioxidant defence system, reduced elimination of pro-oxidant substances and replacement therapy in dialysis patients [4]. This oxidative phenomenon also results from the recruitment of monocytes and macrophages, which are a major source of free radicals and, therefore, mediate the phenomena of autophagy, renal tubular epithelial cells apoptosis, and pro-fibrotic mechanisms. The condition of oxidative stress is closely linked to a pro-inflammatory state in the course of kidney damage, as the pro-inflammatory stimulus increases the response of circulating immune cells, and the redox imbalance simultaneously contributes to the increase in the circulating pro-inflammatory cytokines such as interleukin-6 (IL-6) or tumor necrosis factor-alpha (TNF-α) [22,23,24,25,26,27,28,29]. On the other hand, in uremic patients, mostly on maintenance hemodialysis, in addition to a reactive oxygen species (ROS) overproduction, CKD is associated with a significant impairment of antioxidants, e.g., superoxide dismutase (SOD) and glutathione peroxidase (GPx), thus generating an oxidative stress condition [22].

In patients with CKD, oxidative stress is also related to an increased incidence of anaemia, atherosclerosis, and dyslipidaemia due to alterations in protein and lipid components and reduced clearance of triglyceride-rich lipoproteins. These disorders appear to be related in particular to an alteration in the lipoproteins themselves, with a reduction in LDL and IDL catabolism and in HDL plasma concentrations, as well as an inhibition of lipoprotein lipase (LPL) function [23].

Patients with CKD typically undergo hemodialysis or continuous peritoneal dialysis while awaiting a kidney transplant, and these treatments often trigger an inflammatory response. During dialysis, pro-inflammatory cytokines, uremic toxins, and pro-fibrotic markers are upregulated. Some studies suggest there are no significant differences in kidney damage markers between the two therapies, although the use of synthetic membranes in hemodialysis seems more closely associated with the development of inflammatory processes [30]. Additionally, other factors contribute to the inflammatory state of CKD. Overhydration leads to swelling in the intestinal mucosa and increased permeability of the blood–intestinal barrier, resulting in a “leaky gut” condition, where bacterial toxins and pathogens pass from the gastrointestinal tract into the bloodstream. These factors, common in patients with impaired renal function, also heighten the production of reactive oxygen species (ROS) by respiratory burst enzymes [25]. The gut microbiota–kidney interaction is identified as the “gut–kidney axis” [26]. Many factors contribute to the alteration of the microbiota in CKD patients, such as high levels of ammonia, which lower the pH in the gastrointestinal tract; prolonged colon transit; fluid overload; dietary restrictions, which result in reduced fiber consumption; and medications (e.g., proton pump inhibitors, potassium and phosphate binders, oral iron, and antibiotics). Alterations in the healthy gut microbiota can have many consequences, including intestinal dysbiosis, intestinal barrier dysfunction, and bacterial translocation, leading to increased immune system activation. It has been observed that a reduction in the overall bacterial population and diversity in CKD leads to the generation and widespread accumulation of pro-inflammatory uremic toxins, including indoxyl sulfate (IS), p-cresyl sulfate, ammonia, amines, and trimethylamine N-oxide [22].

The “leaky gut” condition in CKD, characterized by disruption of the intestinal epithelial barrier, also promotes the systemic absorption of these toxins, which are responsible for inflammation, oxidative stress, endothelial damage, and protein energy wasting (PEW). Disruption of healthy microbiota can impair gut immune function and, in the worst case, create a state of “immunoparalysis” with systemic consequences of systemic cytokine activation, uremic toxin accumulation, and endotoxemia [27]. Therapeutic approaches aimed at restoring normal gut microbiota in CKD patients may be useful to ameliorate the inflammatory state, oxidative stress malnutrition, and several comorbidities as well [28]. Taken together, all of the above factors contribute to the onset of chronic inflammation with adverse consequences, most notably poor quality of life and increased mortality, including systemic complications, such as CVD and infectious complications. As in other chronic diseases, targeting different levels of the inflammatory cascade could improve the prognosis of patients with CKD, even in more advanced stages of the disease itself, such as ESRD [29,30,31].

## 4. Pro-Inflammatory Factors in CKD

Elevated levels of cytokines, involved in the innate immune system, are prevalent in CKD patients, and through acute-phase effector proteins, they can control the inflammatory response and mediate some of its downstream effects. In particular, as CKD progresses, these pro-inflammatory molecules exacerbate the signs and symptoms of the uremic syndrome [32].

In this scenario, relevant inflammatory markers in CKD patients are TNF-α, IL-6, IL-1, CRP, adipokines, and adhesion molecules, as well as the ligand CD40, which is implicated in the progression of CKD. CRP is a pentameric protein of hepatic origin that is a reliable biochemical marker of systemic inflammation, as it can also be produced by many inflammatory cells [33]. High levels of CRP are considered a predictor of cardiovascular events, which makes CRP a risk factor for CKD patients [34]. Moreover, elevated levels of CRP are a risk factor also for the common mortality causes in CKD patients in stages 3 and 4 [35]. In fact, in patients with renal diseases, kidney tubular and endothelial cells, as well as macrophages, express high levels of CRP [33]. Several causes are associated with increased CRP in CKD patients [36,37]. One cause is the release of pro-inflammatory cytokines, especially IL-6, which can stimulate the liver to produce CRP [38]. Oxidative stress, which is upregulated in CKD and related to the state of systemic inflammation, also increases CRP levels [39], the elimination of which is impaired by reduced renal clearance, thus favoring its accumulation [33]. In addition, uremia and the retention of uremic toxins due to impaired renal functions increase the inflammatory state, further contributing to the increase of CRP levels [40]. CRP can have two conformational isoforms: the native pentameric CRP (pCRP) and monomeric CRP (mCRP) form, which both exert pro- and anti-inflammatory activity [41]. These two different isoforms of CRP may have different functions under different conditions: pCRP exhibits pro-inflammatory activities on several cell types, such as kidney cells and macrophages, which can lead to renal inflammation and fibrosis. In particular, pCRP from the damaged kidney can promote the infiltration of inflammatory cells and the release of inflammatory factors, such as cytokines, chemokines, and TGF-β1. Although CRP is stable, it can dissociate into the mCRP isoform, exacerbating the progression of inflammation-based diseases through the onset of atherosclerosis and ischemia-reperfusion injury (IRI). Meanwhile, mCRP could limit the amplification of tissue injury by inhibiting renal cell-directed complement activation [33].

The role of CRP in the inflammatory state associated with CKD has been well established using a mouse model of unilateral ureteral obstructive nephropathy (UOO). In this condition, CRP can stimulate NF-κB signaling to induce the expression of monocyte chemotactic protein 1 (MCP-1) and adhesion molecule (ICAM-1) by inducing Nf-kB signaling. This process can lead to macrophage infiltration and renal inflammation, inducing the expression of IL-1 β and TNF-α [42]. This suggests that CRP exerts its pro-inflammatory role in renal injury through direct or indirect activation of NF-κB. The profibrogenic mechanism underlying macrophage infiltration at the site of injury is a key factor in the transition of AKI into CKD, as the macrophage phenotype M2 not only contributes to the repair of the injured tissue but also promotes the fibrotic process. However, it has been shown that macrophage infiltration is also mediated by M1 macrophages, whose polarization is induced by CRP itself [43]. A study involving human transgenic mice affected by CRP has revealed an additional mechanism through which CRP mediates fibrosis and renal inflammation: the activation of Smad3. In kidney damage, Smad3 is involved in TGF-β signaling. In particular, when CRP binds to CD32, Smad3 is activated by the TGF-β1 pathway, as well as the ERK/p38 MAPK-Smad pathway, regulating cell death through the mechanism of Smad3-p27-dependent G1 cell cycle arrest. The incidence of acute kidney injury (AKI) and chronic kidney disease (CKD) is reduced in mice lacking Smad3, suggesting that genetic deletion or pharmacological inhibition of Smad3 may help reduce kidney damage in mouse models of ischemia-induced kidney disease [44]. Modulating CRP could offer a promising therapeutic approach from a clinical perspective. However, the therapeutic potential of anti-CRP treatment in both acute and chronic CKD is still in its early stages, and further studies are needed to establish CRP’s role in this disease.

Another common element in patients with CKD is the elevated plasma level of IL-6 [22,45], one of the most studied cytokines in CKD, which regulates many metabolic and neural processes; it is also well known for its modulation of acute phase proteins and B cells [46]. In CKD, IL-6 is released by renal cells (such as tubular epithelial cells, podocytes, and mesangial cells), endothelial cells, adipose tissue, and lymphocytes under the influence of stimuli such as lipopolysaccharide (LPS), TNF-α, IL-1β, and oxidative stress [47,48]. IL-6 is an interesting molecule in the pathogenesis and progression of CKD as it has both pro- and anti-inflammatory effects since IL-6 is involved in the recruitment of leukocytes, and it can also induce lymphocyte proliferation, as well as their activation and B-cell differentiation [25]. IL-6 also exerts its pro-inflammatory role through increased hypertension, alteration of erythropoiesis, anemia, and upregulation of fibroblast growth factor (FGF23), all of which contribute to increased mortality in patients [49].

In addition to its deleterious role in renal injury, IL-6 produced by renal cells may have an anti-inflammatory effect. In particular, following the signal induced by TNF-α, podocytes secrete IL-6, which induces its immunosuppressive action and, consequently, the recruitment of neutrophils into the endothelium. The pro- and anti-inflammatory action of IL-6 suggests that further studies are needed to clarify its role in acute and chronic diseases through further studies [47]. In particular, in the context of CKD, IL-6 accelerates the progression of CKD and also contributes to its complications, especially CVD [47,48]. This cytokine plays an important role in atherosclerosis, a condition found in dialysis patients and closely associated with the presence of carotid plaques and increased aortic stiffness [50]. IL-6 has been shown to induce endothelial damage also by reducing endothelial expression of nitric oxide synthase (eNOS) and adiponectin and increasing levels of ROS, which would explain the contribution to the development of atherosclerosis itself and vasoconstriction [51,52]. IL-6 can also cause malnutrition by increasing protein catabolism and eating behavior, and indeed, anorexia in patients on hemodialysis or with kidney disease is associated with higher levels of IL-6 [53]. Patients with advanced CKD who develop anemia and concomitant erythropoietin resistance may require erythropoiesis-stimulating agents, such as high-dose erythropoietin. Preclinical findings in animal models suggest a different role between IL-6, its receptor, and its anti-IL-6 receptor in kidney diseases. In fact, neutralization of IL-6 [54] and IL-6 receptor (IL-6R) reduced disease severity, while it has been highlighted that anti-IL-R6 or anti-IL-6 strategies increased the severity of nephritis in the study models [48,55].

IL-1 is a primary pro-inflammatory cytokine with a key role in both acute and chronic inflammation by enhancing inflammatory cell infiltration and increasing the expression of adhesion molecules [56,57,58,59]. Patients with CKD have elevated serum IL-1 levels [60]. Over the years, IL-1 has been hypothesized to underlie many complications in chronic dialysis patients. However, the inflammatory process associated with CKD is known to begin long before the need for chronic dialysis. The inflammasome, a protein structure that causes IL-1 activation, is active in CKD regardless of the etiology.

The primary effects of IL-1 in chronic kidney disease (CKD) are related to mineral and bone disorders [61], but IL-1 also plays a role as a potential mediator of inflammation in heart failure and the cardiovascular complications associated with CKD [62]. It has been suggested that inhibiting IL-1 could reduce cardiovascular risk in CKD patients. In a randomized, double-blind study, 42 adult patients with stage 3–4 CKD were treated with either rilonacept, an IL-1 inhibitor, or a placebo for 12 weeks. The study found that inhibiting IL-1 improved endothelial-dependent dilation (EDD), a key precursor to cardiovascular disease (CVD), in patients with moderate to severe CKD. Specifically, rilonacept treatment was associated with a reduction in systemic inflammation and vascular oxidative stress, as evidenced by lower levels of the oxidative enzyme, NADPH oxidase, and C-reactive protein (CRP), as compared to the placebo group [61].

IL-1 in CKD also induces the expression and production of ICAM-1 by glomerular cells and tubular epithelium, which facilitates the adhesion and infiltration of leukocytes and endothelium into renal tissue and promotes renal fibrosis [63]. It is currently unknown whether IL-1 inhibition in patients with CKD reduces cardiovascular risk in patients with CKD is possible. However, a double-blind, randomized, placebo-controlled, 12-week trial using an IL-1 inhibitor provided the first evidence that IL-1 inhibition improves endothelial-dependent dilation (EDD), a crucial predictor of future cardiovascular events and associated mortality in patients with moderate to severe CKD who do not require chronic dialysis [64]. A clinical trial of canakinumab (a neutralizing antibody against IL-1β) observed a reduction in the rate of cardiovascular events in patients with CKD without modifying renal function, which is clinically relevant, as few therapies have been shown to be effective in reducing the likelihood of cardiovascular events in patients with kidney disease. In the future, it may also be useful to test both the efficacy of canakinumab in patients with end-stage renal disease on dialysis and the efficacy of other anti-inflammatory agents that may be useful in CKD, such as anakinra, an IL-1 receptor antagonist, which can inhibit IL-1β and IL-1α, on which few studies have been conducted [65].

TNF-α is the main regulator of the cytokine cascade and is a potent mediator of the inflammatory response produced by various cells such as macrophages, mesangial cells, and tubular epithelial cells [66] in response to numerous stimuli such as LPS, angiotensin II, calcium-sensitive receptor (CaSR) activation, hypertension, renal failure, glomerulonephritis, diabetic nephropathy, and interstitial tubular nephritis [67]. Kidney-resident dendritic cells have been shown to be particularly involved in the secretion of TNF-α in patients with early renal ischemia-reperfusion injury [68]. In CKD, TNF-α is implicated in the progressive loss of kidney function, and its production is directly related to the severity of renal injury [69,70,71].

Caspases pathway, NF-κB pathway, and mitogen-activated protein kinase (MAPK) pathway are the primary mechanisms activated by TNF-α. The pleiotropic effects of TNF-α are mediated by two tumor necrosis factor receptors (TNFR1 and TNFR2), which activate downstream signaling pathways. Binding of TNF-α to TNFR1 enhances inflammation and tissue damage, whereas binding to TNFR2 is potentially involved in the regulation of immunity, as well as the regeneration of tissue [72]. TNF-α exerts various effects on cells and is also associated with ROS in a variety of cells, including mesangial cells [73], leading to the impairment of the barrier function of the glomerular capillary wall [74]. A member of the TNFR superfamily, commonly referred to as cluster of differentiation 40 (CD40), and its natural ligand, CD40L, are mediators involved in the inflammatory process. In fact, circulating levels of the CD40 receptor and its ligand are closely related to kidney damage. This is evidenced by the fact that CD40 is expressed on the membrane of different types of kidney cells, allowing the interaction between immune and renal cells during the progression of kidney damage. CD40 and CD40L are also expressed by activated T and B cells, but CD40L also exists in a soluble form (sCD40L) [75,76]. Elevated levels of circulating sCD40L are associated with a higher risk of cardiovascular morbidity and mortality, particularly in patients on hemodialysis (HD) [77,78]. In addition, plasma levels of soluble CD40 receptor (sCD40), an isoform of the CD40 receptor, predict the progression of renal dysfunction in patients with CKD [79]. The involvement of CD40 in immune cells has been associated with local kidney inflammatory state, and CD40/CD40L signaling in various renal cell types has been implicated in mediating glomerular permeability, interstitial inflammation, and fibrosis in kidney disease. Several factors in renal cells can modulate CD40 expression. In particular, the binding of CD40 to its ligand plays a key role in the interaction between immune cells and local renal cells in CKD [23]. Therefore, targeting CD40 may be a promising approach and a potential therapeutic method for the treatment of kidney disease. TNF-α is involved in the release of other mediators of the inflammation in CKD, such as IL-1, prostaglandins, oxygen radicals, NO, and platelet-activating factor [80,81] by activating the nuclear factor kappa B (NF-kB) and MAPK signaling pathways. The therapeutic use of anti-TNF monoclonal antibodies, such as the TNF inhibitor, etanercept, is currently approved for the treatment of autoimmune diseases, such as Crohn’s disease and rheumatoid arthritis. An in vivo study investigated the modulation of TNF levels by etanercept and the consequences of its inhibition on the progression of renal damage. In particular, this study in rats with adenosine-induced chronic renal failure showed that etanercept was able to significantly reduce serum TNF-α levels within 2 to 4 weeks of treatment. In addition, etanercept administration can reduce interstitial fibrosis, tubular atrophy, and inflammatory infiltrate induced by adenosine administration after 2 weeks. However, etanercept did not prevent renal fibrosis that occurred 4 weeks after adenosine administration [80]. Given the importance of TNF signaling pathways in the progression of CKD, further studies on the effects of TNF-targeted drugs would be useful. In addition, specific inhibition of TNFR1 or TNFR2 may help to elucidate the pro-inflammatory and immunomodulatory roles of each of the two receptors [82].

In recent years, adipokines have been studied for their role in CKD. Adipokines are proteins released by adipose tissue that play an important role in cell signaling and regulation of metabolism and inflammation, and their dysregulation contributes to the development of disorders associated with obesity, cancer, kidney damage, and cardiovascular disease. White adipocytes in adipose tissue are able to convert surplus energy in triglycerides and secrete several adipokines, including leptin and adiponectin, and TNF-α and IL-6. Brown adipocytes, on the other hand, store energy in the form of small lipid droplets and secrete IL-10, which has beneficial effects on energy expenditure, glucose homeostasis, lipid metabolism, and the inflammatory process [83]. Among the adipokines, leptin and adiponectin are associated with CKD. The pro-inflammatory effect of leptin is an increase in cytokines, such as TNF-alpha, IL-1, and IL-2, which causes an increased incidence of insulin resistance, atherosclerosis, and endothelial dysfunction. Leptin is involved not only in the release of inflammatory cytokines but also in the proliferation of CD4+ T cells and the inhibition of neutrophil chemotaxis, leading to an increased risk of persistent CKD infections [84]. Low levels of adiponectin are a strong risk factor for cardiovascular disease and even hypertension in patients with CKD; in fact, higher levels of adiponectin have been reported to be negatively associated with progression to renal failure [85]. Endothelial dysfunction may also be related to increased synthesis of intercellular adhesion molecules (ICAM)-1, which, together with adiponectin, is indicative of albuminuria. Moreover, the inhibition of ICAM-1 during renal damage is linked to the anti-inflammatory activity of adiponectin [86]. In fact, increased ICAM-1 levels are associated with the accumulation of immune cells in the renal tissue and with both loss of renal function and fibrosis development [87]. Adhesion molecules are important players that, once activated, they are released from the surface of endothelial cells [88] and are promising biomarkers reflecting endothelial activation and vascular inflammatory status [89]. The expression of cell adhesion molecules can be altered by several factors, including hypertension [90] and immunosuppressive therapy [91,92].

In pre-dialysis patients, ICAM-1 is also associated with CRP [93]. Although high levels of VCAM-1 molecules are a predictor of cardiovascular events in patients with CKD, there is little evidence to support this increase in atherosclerotic vascular tissue during CKD. However, a 2021 study on arterial smooth muscle cells showed that VCAM-1 expression in arterial smooth muscle cells was more significant in patients with advanced kidney disease than in those with normal kidney function. This suggests that VCAM-1 could be a cardiovascular prognostic biomarker in patients with CKD [90]. In addition to cytokine production and tissue damage, inflammasome activation in CKD can induce cellular stress responses, including oxidative stress and mitochondrial dysfunction [21]. ROS generated during inflammasome activation contributes to renal oxidative stress, which further exacerbates inflammation and tissue damage in the kidney. However, the relationship between the inflammasomes and chronic renal failure remains controversial. Activation of the inflammasome induces the cleavage and the subsequent activation of caspase-1. This mechanism can regulate the maturation and release of pro-inflammatory cytokines [84].

One of the most studied inflammasomes in relation to CKD is the pyrin domain-containing NOD-like receptor family 3 (NLRP3) inflammasome [83,94]. NLRP3 is a cytosolic sensor protein that forms a multiprotein complex in response to various danger signals, such as pathogen-associated molecular patterns (PAMPs) and damage-associated molecular patterns (DAMPs) [95,96,97].

In CKD, dysregulation of the NLRP3 inflammasome has been implicated in the progression of renal injury and inflammation. Uremic toxins, such as IS, have been shown to induce NLRP3 activation and IL-1β production in renal cells [95]. Furthermore, the inflammasome is activated in dialysis patients with uremia, and this condition is associated with mitochondrial dysfunction, which is essential for NLRP3 inflammasome activation. In this context, excess ROS overstimulates the inflammasome, leading to severe tubulointerstitial damage and renal tubular cell apoptosis [98]. A 2021 study attributed poor NLPR3 activation to the presence of IS, suggesting that a lack of activation of the inflammasome itself causes a reduction in responsiveness to pathogens, with downregulation of caspase-1 and IL-1β levels. This concept is supported by the fact that CKD presents as a state of persistent low-grade inflammation, with continued exposure to pro-inflammatory agents reducing the expression of the inflammasome as well as NF-kB [99]. Macrophages also play an important role in modulating NLPR3 and maintaining renal homeostasis. In fact, they are able to activate NLRP3 through the NF-κB signaling pathway following noxious stimuli. This complex stimulates the activation of caspase-1, resulting in the production of pro-inflammatory cytokines like IL-1β and IL-18. Macrophages in this state polarise from an M1 phenotype, which causes renal inflammation, pyroptosis, and renal fibrosis, to an M2 phenotype, which is decisive in kidney repair and regeneration processes. Previous studies in murine peritoneal macrophages have shown that IS is able to induce an increase in the expression of pro-inflammatory mediators, such as cyclooxygenase (COX-2), inducible nitric oxide synthase (iNOS), as well as the pro-apoptotic protein Bax, an index of macrophage dysfunction [100]. Given the important role of macrophages in the inflammatory process in kidney disease, inhibiting macrophage recruitment and activation may be a good therapeutic strategy for kidney damage, including targeting the inflammasome itself [101].

In patients with CKD, kidney damage is linked to a decrease in the protective ability of antioxidant systems and a disruption in the nuclear translocation of nuclear factor (erythroid-derived 2)-like 2 (Nrf2). Downregulation of Nrf2 in CKD leads to increased renal fibrosis, tubular damage, and worsening of the disease. In addition, reduced expression of Nrf2 in hemodialysis patients contributes to defective mitophagy, overproduction of ROS, and dysregulation of cellular metabolism, exacerbating CKD [102]. As shown in an in vitro study on intestinal epithelial cells, pro-inflammatory stimuli, such as LPS or IS, are able to influence antioxidant defense cell systems, such as HO-1, NAD(P)H quinone oxidoreductase 1 (NQO1), and SOD, causing a state of oxidative stress not only at the renal level but also at the systemic level and particularly in the gut, which is considered a primary source of inflammation in CKD [103].

**Figure 1:**
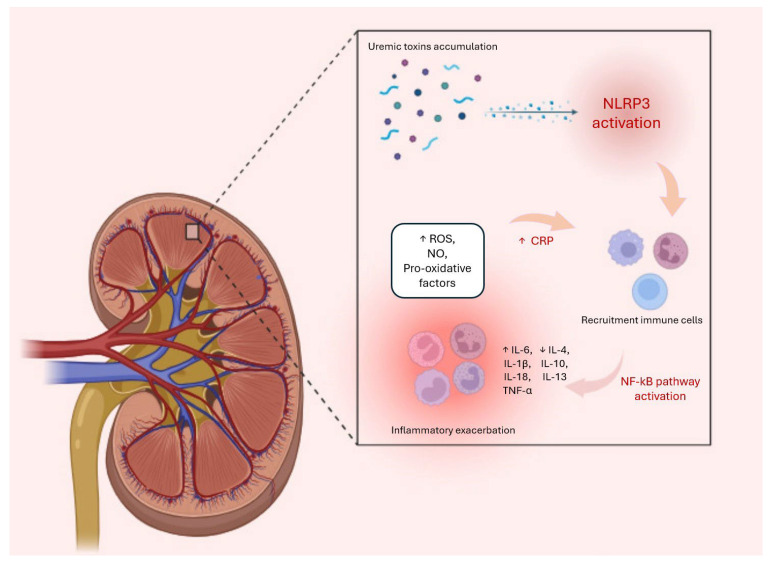
During the progression of CKD, uremic toxins play a significant role in amplifying the inflammatory response by activating NLRP3, which triggers cellular stress responses. These include increases in nitric oxide (NO), reactive oxygen species (ROS), and levels of pro-inflammatory cytokines, such as IL-1, IL-1β, and TNF-α. Meanwhile, the production of pro-inflammatory cytokines and chemokines promotes the recruitment of leukocytes into the kidney. This process leads to an exacerbation of the inflammatory system, also represented by an increase in CRP levels and a decrease in anti-inflammatory cytokines, such as IL-10. The combination of these processes is responsible for the onset of various systemic complications, including fibrosis, atherosclerosis, anaemia, mineral disorders, and cardiovascular events.

The renin-angiotensin-aldosterone system (RAAS) is another key player involved in the promotion of inflammatory processes in CKD through several mechanisms. Specifically, angiotensin II, a key component of the RAAS, exerts direct pro-inflammatory effects by stimulating the production of cytokines, such as IL-6 and TNF-alpha, along with adhesion molecules and chemokines that facilitate leukocyte recruitment and inflammation in the kidneys [104,105]. Indeed, the RAAS interacts with several inflammatory pathways, including NF-Κb and mitogen-activated protein kinase (MAPK) signaling pathways, to amplify inflammatory responses and the expression of pro-inflammatory mediators in kidneys [106,107]. In addition, angiotensin II can modulate immune cell activation and promote the differentiation and activation of immune cells, such as macrophages and T lymphocytes, which further contribute to inflammation and renal damage in CKD [75]. All these processes are schematized in Figure 1 below:

## 5. Available Treatments

The management of CKD is mainly directed to prevent the disease progression to ESRD and other complications associated with CKD, such as cardiovascular disease (CVD), hypertension, and type 2 diabetes. Despite this, drugs employed in CKD treatment can induce severe side effects in patients. For example, administration of renin–angiotensin–aldosterone system inhibitors (RAASi) is suggested to manage hypertension, but patients on RAASi therapy have to occasionally discontinue their treatment or reduce dosage because RAASi can induce hyperkalaemia [108,109]. In CKD, treatment is fundamental to consider the management of inflammation since CKD is associated with high levels of pro-inflammatory factors. In fact, investigations have been conducted on the effectiveness of TNF-α inhibitors, such as etanercept, which showed a significant decrease in rat serum TNF-α levels, even if unsuccessful in preventing renal fibrosis [110]. Also, statin therapy is contemplated in therapies for CKD patients, especially to reduce the risk of CVD [111], addressing key points of inflammation. In fact, statins can induce inhibition of the NLPR3 inflammasome, IL-1β production; decrease serum c-reactive protein (CRP) and ROS levels; avoid NF-κB accumulation; and increase catalase and superoxide dismutase activity [112]. However, statin drugs are associated with musculoskeletal side effects, such as rhabdomyolysis, which can induce tubular injury and ischaemia [113] and increase the levels of creatine kinase, which exacerbate renal impairment with myoglobinuria and electrolyte alteration [114].

Recently, results were reported from a phase 2 trial, the RESCUE study (Reduction in Inflammation in Patients with Advanced Chronic Renal Using Antibody-Medicated IL-6 Inhibition), on patients with advanced CKD who showed high C-reactive protein levels, which investigated the anti-inflammatory activity of ziltivekimab, a human anti-IL-6 monoclonal antibody. Subjects treated with ziltivekimab were shown to have a reduced need for erythropoiesis-stimulating agents. Although these results with ziltivekimab do not provide evidence of a direct benefit of Ziltivekimab on renal function, it is believed that treatment with ziltivekimab may lead to a reduced risk of CVD in patients with CKD. Although the results of the study are not enough to affirm the direct benefit of ziltivekimab on renal function, it is believed that treatment with ziltivekimab may lead to a reduced risk of CVD in patients with CKD. Targeting IL-6 in CKD appears to be a promising avenue, as it has a key role in the progression of renal disease, as well as in associated complications. However, the IL-6 inhibitors currently approved for clinically inhibit IL-6 release in CKD therapy are not promising at this time [115].

Preclinical findings in animal models suggest a different role between IL-6, its receptor, and its anti-IL-6 receptor in kidney diseases. In fact, neutralization of IL-6 [55] and IL-6 receptor (IL-6R) reduced disease severity, while it has been highlighted that anti-IL-R6 or anti-IL-6 therapeutical approaches can worsen nephritis in the study models [116]. CKD patients are required to follow a diet low in protein and sodium in order to control renal dysfunction, hypertension, and complications associated with CKD. In fact, to prevent diabetes and cardiovascular complications, changes in patients’ eating habits are suggested, such as introducing the Mediterranean diet [117] or PUFA supplementation, which results are beneficial in decreasing oxidative stress and cytokine levels involved in inflammatory pathways in CKD [118]. A healthy diet is obviously associated with a correct lifestyle, with reduced alcohol consumption, smoking, increased physical activity, and specific dietary behaviours for each stage of the disease [119]. Very often, CKD patients are subject to polypharmacy, and concomitant use of drugs such as antibiotics can have a negative impact on the gut microbiota. For this reason, patients themselves are advised to follow a diet rich in fibre, which promotes the growth of saccharolytic SCFA-producing bacteria and concomitant reduction of intestinal-derived uremic toxins; this approach may in fact preserve the integrity of the intestinal barrier and mucus production, thereby reducing inflammation [120]. Another therapeutic strategy is the supplementation of prebiotics, probiotics, and symbiotics; however, studies are limited by small sample sizes, variations in doses, strains, outcome measures, and duration of intervention. Agents such as activated charcoal (e.g., AST-120) have also been shown to significantly reduce oxidative stress and inflammation and IS and p-CS concentrations in animal models. [121]. Despite the benefits of a healthy diet and available drug therapies, more research needs to be conducted, considering that this kind of intervention can only slow the progression of ESRD. One target of research in this context could be strategies aimed at fostering the development of a symbiotic microbiota to further investigate the effect on CKD progression. However, therapeutic choices could be influenced by an accurate and early diagnosis in order to intervene early in the progression of the disease; in this regard, genetic tests could help in identifying the aetiology and therapy to be chosen. This approach would also make it possible to have a personalised therapy for each patient that can take into account the risk factors, lifestyle, environmental factors, and co-morbidities and drug tolerance associated with that same patient. Pharmacogenomic studies can provide important information on drug selection and dosage choice to reduce side effects, an important aspect for patients with reduced renal function. Despite its potential, personalised medicine has not yet been widely adopted in the clinical setting due to high costs and problems with the accessibility of genetic testing and associated patient data [122].

## 6. Inflammatory Resolution Mediators

To prevent the transition from acute to chronic inflammation, it is essential to regulate the inflammatory response. Resolution of inflammation is a spatially and temporally controlled process characterised by reduction of leukocyte infiltration, apoptosis of neutrophils at the site of injury, and efferocytosis by macrophages of apoptotic cells and debris without systemic host immune suppression. The events involved in the resolution process were first presented by Robbins and Cotran [106] and complemented by Savill and collaborators [107]. They suggested that in the resolution phase, the inflammatory exudate is reorganised, with macrophages engaging in phagocytic activity to remove dead cells and debris caused by inflammation. Later, other researchers expanded on this idea, identifying a possible role for lipid mediators derived from omega-6 (n-6) and omega-3 (n-3) polyunsaturated fatty acids; in 2002, Serhan and co-workers studied inflammatory cellular exudate to elucidate the mechanism of inflammatory response in vitro and in animal models [123,124], defining the resolution of inflammation as a dynamic and hierarchical process in which lipid-derived mediators play a central role.

**Figure 2:**
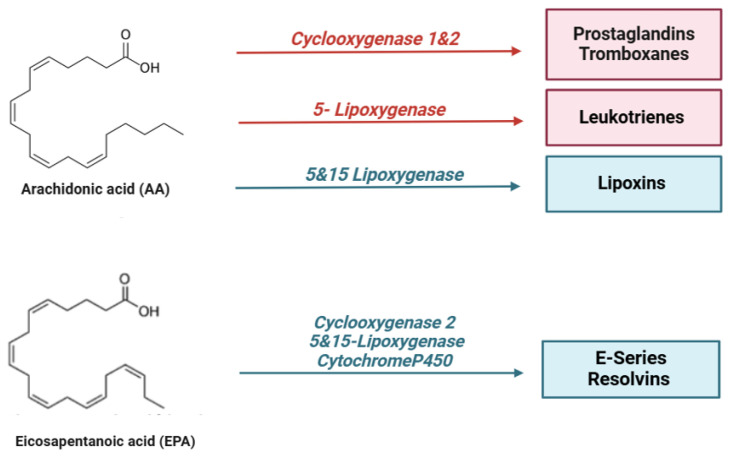

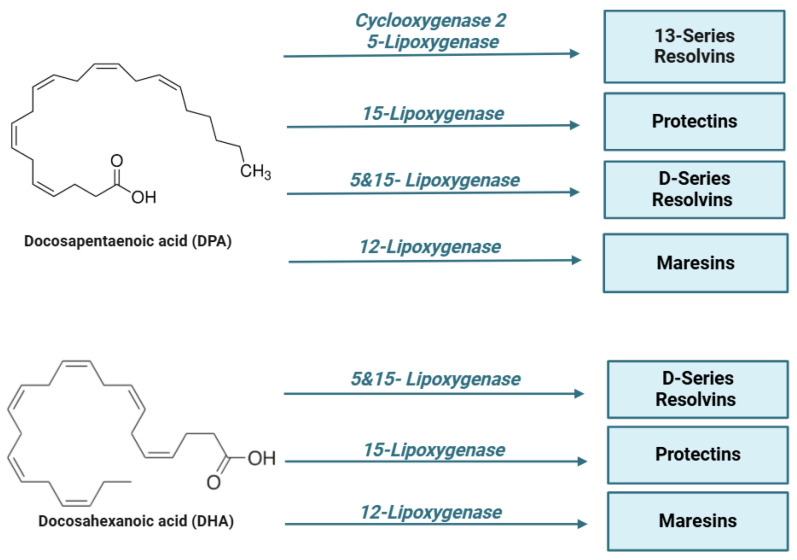
Lipid mediators are derived from ω-6 and ω-3 polyunsaturated fatty acids (PUFAs). During the early initiation phase of inflammation, cyclooxygenase (COX) and lipoxygenase (LO) enzymes begin the conversion of the ω-6 polyunsaturated fatty acid, arachidonic acid, into a series of bioactive lipid molecules, such as prostaglandins (PGs), thromboxanes (TXs), and leukotrienes (LTs). During the resolution phase of inflammation, specialized pro-resolving lipid mediators (SPMs) are synthetized. Arachidonic acid produces lipoxins (LXs), while eicosapentaenoic acid (EPA), n-docosapentaenoic acid (DPA), and docosahexaenoic acid (DHA) produce lipid mediators, including resolvins (RVs), protectins (PD), and maresins (MaRs). Of note, the mouse 15-lipoxygenase carries both 12- and 15-lipoxygenase activity.

In particular, specialized pro-resolving lipid mediators (SPMs) are endogenous bioactive mediators that stimulate self-limited innate immune responses, kill and clear microbes, and protect organs. Compared to most anti-inflammatory agents, SPMs show potent resolving effects in various animal models of disease; therefore, these molecules, together with their receptors, represent interesting targets for the control of inflammation [125]. In the early stages of inflammation, there is localized production of pro-inflammatory cytokines (such as TNF, IL-1β, and IL-6) and eicosanoids, which facilitate the recruitment of neutrophils to the site of tissue damage. However, for the resolution process to be effective, it is crucial to halt granulocyte recruitment and simultaneously promote the recruitment and differentiation of macrophages from a pro-inflammatory to an anti-inflammatory phenotype [126]. This macrophage polarization stimulates efferocytosis, leading to the production of specialized pro-resolving mediators (SPMs), which contribute to restoring vascular integrity and regenerating damaged tissues [127]. SPMs are classified into different subgroups according to their origin and structures, as shown in the figure below (Figure 2):

Therapeutic strategies that promote the resolution of inflammation offer a promising approach in the management of CKD. The following is a summary of preclinical and clinical studies in experimental models of kidney disease, which suggest that ω-6 and ω-3 polyunsaturated fatty acids and derivatives are useful in preserving renal function.

### 6.1. Arachidonic Acid Derivatives in CKD

Arachidonic acid (AA) is a ω-6 polyunsaturated fatty acid found in phospholipid form in the cell membrane [128]. In response to a cell-damaging stimulus, arachidonic acid (AA) is released from phospholipids by the action of phospholipase A2 and phospholipase C in its free form. This free AA serves as a precursor for pro-inflammatory bioactive mediators through three main metabolic routes: via the cyclooxygenase (COX) pathway, AA is converted into prostaglandins (PGs) and thromboxanes (TXs); through the lipoxygenase (LOX) pathway, leukotrienes (LTs) and lipoxins (LXs) are produced; additionally, AA generates epoxyeicosatrienoic acids (EETs) or hydroxyeicosatetraenoic acids (HETEs) via the LOX pathway. Together, these metabolites, also known as eicosanoids, play a central role in modulating the inflammatory process [129]. The metabolism of arachidonic acid (AA) is involved in various physiological and pathological processes in the body. AA affects the fluidity and permeability of cell membranes, regulates platelet function, and modulates immune system activation. Additionally, AA and its metabolites play a crucial role in the primary mechanisms contributing to chronic kidney damage by influencing glomerular and tubular function, podocyte pathophysiology, and the progression of renal fibrosis. In vitro experiments on human mesangial cell cultures incubated with arachidonic acid (AA), docosahexaenoic acid (DHA), and eicosapentaenoic acid (EPA) demonstrated that AA could counteract the angiotensin II-induced upregulation of TGF-β, fibronectin 1 (FN1), connective tissue growth factor (CTGF), and collagen IV gene expression, thus activating the mechanisms responsible for renal damage. Treatment of the same cells with EPA or DHA suppressed the upregulation induced by Ang II and AA, suggesting that the balance between different omega-3 and omega-6 fatty acids may negatively or positively affect kidney damage [127]. Furthermore, in immortalised human podocyte cultures, AA treatment activated protein kinase A, which promotes the activation of the adaptor protein c-Abl and the phosphorylation of nephrine, which in its phosphorylated form mediates podocyte survival and function. When dephosphorylated, nephrine releases c-Abl, which accelerates actin and cytoskeletal remodeling, leading to podocyte lesion formation, a critical event in kidney injury [130].

Kidney damage is closely linked to the production of prostaglandins. A well-established connection exists between prostaglandins (PGs) and tubulointerstitial damage, as well as glomerulonephritis. PGE2, the main product of the COX-2 pathway in the kidney, is found to be elevated in conditions such as diabetic nephropathy [131] and plays a crucial role in renal hemodynamics, renin release, and renal tubular sodium/water reabsorption. In contrast, PGI2 and PGE1 help relax blood vessels and protect renal tissue from hypoxia-induced damage. Hydroxyeicosatetraenoic acids (HETEs), which are formed from arachidonic acid (AA) via the LOX pathway, can activate PPARγ in macrophages, induce apoptosis of tubulointerstitial cells, and contribute to inflammation and renal tubular fibrosis [128]. HETEs also play a role in regulating renal ion transport during renal inflammation. Specifically, 20-HETE modulates cell proliferation, angiogenesis, and serves as a key regulator of renal function [132]. However, not all metabolites derived from AA metabolism have pro-inflammatory effects, especially in renal damage; lipoxins, which are derived from the same precursor, have a pro-resolving action.

#### Lipoxins

Arachidonic acid (AA). Among all the SPMs that promote the resolution of inflammation, the LX lipoxins were the first to be identified [133]; LXs were isolated from human leukocytes by Serhan and co-workers, and they named these mediators “lipoxins” because they are “lipoxygenase interaction products” [134].

LX, together with their synthetic analogues, showed anti-inflammatory and pro-resolving effects on in vitro and in vivo models [135]. However, although the native lipoxins, LXA_4_ and LXB_4_, have anti-inflammatory and pro-resolving effects, they are rapidly metabolically inactivated via PG dehydrogenase-mediated oxidation and through reduction reactions, which limits their potential application in therapy development [136]. As a result, many LX analogues have been chemically synthesized to prolong the half-life of LX in vivo, and some of these analogues show better stability and more potent activity than native LXs [137]. LXs have several actions in the inflammatory process: they inhibit the migration of neutrophils across the endothelium and their interactions with epithelial cells. Moreover, LXs inhibit neutrophils to release the toxic products of their granules [138] and the formation of peroxynitrite at the site of inflammation, lowering the levels of nuclear factor κB (NFκB) and activator protein-1 (AP-1) in the nucleus, thereby reducing the production of IL-8 [139]. In addition, LXA_4_ production is involved in the inhibition of superoxide release and colonocyte apoptosis, which results in pro-resolving during the inflammatory process [140]. These anti-inflammatory effects are mediated by lipoxins through signals activated by binding to the high-affinity G protein-coupled lipoxin A4 receptor (ALX)/formyl peptide receptor (FPR2) [141]. Other receptors that interact with LX include G protein-coupled receptor 32 (GPR32), the Aryl hydrocarbon receptor, estrogen receptor [142], and the high-affinity cysteinyl leukotriene receptor [143]. LXs have been shown to be pro-resolving in CKD and in various associated CKD complications. Specifically, lipoxins exert their effects by modulating various signalling pathways in the kidney. For example, in the presence of lipoxin in epithelial cells, an increase in the level of let-7c miRNA is observed [144]. Fibrotic damage can also be negatively regulated by LXA_4_ through modulation of the transcription factor EGR-1, which regulates the transcription of the pro-inflammatory cytokines, IL-2 and TNFα, in T cells. The transcriptional repressor NAB1 downregulates EGR1 expression. In neutrophils, LXs have been shown to upregulate NAB1 expression and thereby reduce EGR1 levels [145,146]. LXs and their analogues have also proven effective in treating acute renal failure in dendritic cell mouse models. When mice with renal failure were treated with lipoxin, there was an increase in the mRNA levels of cytokine signaling suppressors (SOCS-1, 2), which bind to other cytokine receptors and inhibit their pro-inflammatory effects [147]. However, the precise impact of LXs on renal dendritic cells remains to be fully understood. In recent years, the role of LX in CKD associated with complications such as diabetes has also been investigated: in a 2018 study using mice with diabetes-associated CKD as an in vivo experimental model, LXA_4_ was shown to suppress diabetes-induced kidney damage, as evidenced by the reduction in albuminuria. LX also attenuated glomerular dilatation and mesangial matrix deposition [146]. The mice were fed a high-fat diet for 3 months to induce this pathological condition and were treated with LX between the fifth and twelfth week of the study. They stimulated macrophage polarization toward a resolution phenotype. In particular, LXA_4_ attenuated the diet-induced increase in the proportion of pro-inflammatory M1 macrophages and partially restored the population proportion of anti-inflammatory M2 macrophages. LXA_4_ treatment also resulted in an obesity-induced reduction in the expression of the pro-inflammatory cytokine TNF-α and partially restored adiponectin levels that had been reduced by the diet. LXA_4_ was also shown to significantly attenuate obesity-induced albuminuria and H_2_O_2_-mediated oxidative damage, indicating protection against kidney damage by attenuating free radical production [148]. LX may, therefore, represent a therapeutic strategy for both CKD and its associated inflammatory complications.

### 6.2. Eicosapentenoic Acid Derivatives in CKD

Eicosapentaenoic acid (EPA) is an omega-3 polyunsaturated fatty acid known as an anti-hyperlipidemic agent, which is known to have antioxidant or anti-inflammatory effects in cardiovascular disease and atherosclerosis; it also appears to reduce levels of pro-inflammatory cytokines and chemokines [149]. Serum EPA levels in patients with kidney disease are due to dialysis treatment, inflammation, and associated metabolic changes, and this deficiency is negatively correlated with cardiovascular events. Under normal conditions, ω-3 PUFA modulates lipid metabolism, improves cardiovascular parameters, reduces inflammation and oxidative stress, and is associated with a reduction in progression to end-stage renal disease. Interestingly, this effect has been seen to a greater extent in patients undergoing dialysis, while studies demonstrating the effect of supplementation of these compounds in patients not undergoing renal replacement therapy are still needed [150].

In this context, the National Kidney Foundation Kidney Disease Outcomes Quality Initiative (NKF KDOQI) advises incorporating food sources rich in omega-3 polyunsaturated fatty acids (PUFAs) into the diet at least twice a week.

Three trials involving more than 800 hemodialysis patients suggest that EPA therapy may improve clinical outcomes in these patients, in particular, reducing mortality related to cardiovascular events. Among these studies mentioned above, in particular, a randomized, multicenter, double-blind, controlled trial involving 206 patients carried out by Svensson and co-workers showed that the treatment of chronic hemodialysis patients with purified EPA + DHA (daily EPA dose less than 1 g/day) did not lead to a significant reduction in mortality related to cardiovascular events but did result in a significant reduction in the incidence of secondary myocardial infarction. The authors hypothesize that the reduction in myocardial infarction may be due to the anti-inflammatory antithrombotic or antioxidant effects of omega-3 PUFAs [151]. EPA has also been shown to reduce lipotoxicity at the level of renal proximal tubular cells (PTEC) in obesity-related kidney diseases. When there is lipid overload at the cellular level due to suppression of the autophagic system, lipids accumulate, resulting in renal lipotoxicity. EPA supplementation in mice fed a high-fat diet (HFD) reduced lipotoxicity in PTECs (reduction in lysosomal phospholipid accumulation, inflammation, and fibrosis). This suggests a potential use of EPA in complications associated with kidney injury [152].

#### 6.2.1. Resolvins

Resolvins are metabolites produced from the ω-3 polyunsaturated fatty acids, DHA, EPA, and DPA through enzymatic processes catalyzed by lipoxygenases (LOXs), cytochrome P450s (CYP450s), and cyclooxygenases (COXs) [153]. Resolvins (RVs) are categorized into two primary classes: E-series resolvins, which are derived from eicosapentaenoic acid (EPA), and D-series resolvins, derived from docosahexaenoic acid (DHA). E-series resolvins (RvE1 and RvE2) are produced by 5-lipoxygenase or COX-II in vascular endothelial cells. These resolvins can also be acetylated by aspirin to form aspirin-triggered resolvins (AT-Rv) [154,155,156].

#### 6.2.2. E-Series Resolvins

E-series resolvins are currently in clinical trials for eye, lung, kidney, skin, and bowel diseases [157]. RvE1 at nanomolar concentrations has been shown to reduce neutrophil infiltration into inflammatory sites by 50–70% in a TNF-α-induced dorsal air pouch model of inflammation. RvE1 can also be metabolized through 12-oxo-dehydrogenation, producing a biologically inactive product that may serve as a potential biomarker for resolving activity [158]. The anti-inflammatory effects of RvE1 have been demonstrated in a variety of pathological settings; RvE1 reduces IL-12 production by dendritic cells [159] and, in the skin, reduces contact hypersensitivity [160]. RvE1 and RvE2 are potent stimulators of IL-10 and phagocytosis [161].

However, a study reported that RvE1 can also exert direct antifibrotic effects in kidney disease: in a model of unilateral ureteral obstruction (UUO)-induced tissue fibrosis, RvE1 can inhibit fibroblast proliferation in vivo and in vitro. Indeed, administration of RvE1 to mice significantly reduced the accumulation of α-smooth actin myofibroblasts (SMA) and IV collagen deposition over six days. Moreover, at 2–4 days after UUO induction, resolvin administration inhibited the proliferation of myofibroblasts [162]. Therefore, further studies on the effect of RvE1 may be useful to identify the potential antifibrotic effect of this resolvin in renal injury.

### 6.3. Docosohexaenoic Acid Derivatives in CKD

DHA is a long-chain, highly unsaturated omega-3 (N-3) fatty acid and is also known as the precursor of maresins, protectins, and D-series resolvins, which attenuate inflammatory disorders by promoting the resolution of inflammation. DHA is crucial in communication mechanisms, membrane structure and function, synthesis of lipid mediators, cell signaling, and gene expression [163]. DHA is effective in counteracting the chronic inflammatory state found in patients with CKD. In fact, a 2018 study demonstrated the combined effects of n-3 fatty acids (EPA and DHA) and CoQ supplementation on neutrophil release in patients with CKD. In a double-blind controlled study on 85 CKD patients, supplements of n-3 FA (4 g) or CoQ (200 mg) or control (4 g olive oil) have been randomly daily administered for 8 weeks. N-3 FA results to have a potential role in limiting the inflammation since patients who received N-3 FA supplementation exhibited higher levels of LTB_5_ and SPMs released by neutrophils and lower plasm levels of MPO. These findings offer additional insight into the mechanisms through which omega-3 fatty acids (n-3 FAs) may help reduce low-grade inflammation in patients with CKD [164].

17-HDHA is a metabolite of DHA and a precursor of protectins (PD1), which has been shown to be clinically important in a number of immune mechanisms involved in the progression of kidney disease [165,166]. The crucial role of DHA in CKD was demonstrated using an in vivo experimental model in which rats underwent nephrectomy, which resulted in increased urinary albumin excretion, reactive oxygen species, inflammation, and tubulointerstitial fibrosis. Rats on DHA supplementation exhibited an improvement in renal dysfunction and fibrosis by showing higher levels of DHA, EPA, and their metabolites and decreased levels of pro-inflammatory cytokines and cells in the renal inflamed site. Moreover, rats on DHA supplementation exhibited lower IS levels in the kidney, which results are particularly interesting since IS is a protein-bound uremic toxin. In fact, the authors suggested a potential role of DHA as a competitor for IS binding. Although further clinical trials are needed, a potential suppression of the CKD progression and protection against tubular injury could be realized by administrating DHA in order to reduce the levels of PTECs while enhancing IS-induced oxidative stress [167].

DHA appears to slow the progression of kidney failure by reducing IS accumulation and associated oxidative stress. A DHA diet appears to suppress fibrosis in the kidney, as shown in a recent study from 2023. The authors had previously studied the effect of a DHA diet, which was demonstrated in the following study; after rats were nephrectomised for 4 weeks, a DHA diet was administered, which attenuated urinary excretion of albumin, ROS levels in the kidney, and plasma IS and TNF- α levels, which were increased 4 weeks after nephrectomy. The authors also highlighted the role of metabolites derived from DHA (protectin D) and EPA (18-HEPE) in reducing inflammation, oxidative stress, and fibrosis 4 weeks after nephrectomy [168].

In 2022, Kobayashi S. et al. conducted an initial study in CKD cats that were orally administered fish oil enriched with DHA in liquid form for 28 days [169]. Although this study has a limitation regarding the number of animals involved, it suggests a beneficial action of DHA in reducing urinary SDMA, UPC, and NAG levels, parameters of renal function. In addition, DHA appears to have reduced arachidonic acid levels in the same study, and consequently, the authors hypothesised that docosanoids, i.e., bioactive lipids converted by DHA from oxidative enzymes in vivo, may antagonise inflammatory eicosanoids, thus contributing to the kidney-protective effect.

A 2017 study had also shown that DHA can inhibit TGFβ1-stimulated fibroblast activation; these cells play an essential role in the production of extracellular matrix and the progression of renal fibrosis. In the study, this effect was demonstrated in NRK-49F cells and treated for 30 min with DHA and subsequently with TGFβ1 for 12, 24, and 48 h. TGFβ1, at a concentration of 2 ng/mL, stimulates NRK-49F cell activation, resulting in increased α-SMA, fibronectomy, and type I collagen expression. DHA significantly reduced TGFβ1-induced fibroblast activation in a time- and dose-dependent manner, and these data suggest a positive action in modulating renal fibrosis [170].

#### 6.3.1. Maresins

Maresins (MaRs) are lipid mediators synthesized by macrophages from DHA through the action of human 12-lipoxygenase (12-LOX). These mediators have anti-inflammatory and pro-resolving properties, which may support tissue regeneration and potentially serve as therapeutic agents in chronic inflammatory diseases [171]. MARs are thought to have a protective role since they can inhibit the macrophage function [172]. In fact, MaRs can release several substances, which probably exert a pro-resolving action by enhancing phagocytosis and efferocytosis when human macrophages are incubated with MaRs. Additionally, MaRs promote tissue regeneration by downregulating pro-inflammatory cytokines, such as IL-1β, IL-6, and TNF-α, thus facilitating the resolution of the inflammatory process [173]. MaR1, in particular, has been shown to limit neutrophil infiltration and reduce the production and release of CXCL1, a key chemokine involved in neutrophil recruitment. Furthermore, MaR1 promotes neutrophil apoptosis, helping to resolve the inflammatory response [174].

MaRs also play a crucial role in the early stages of kidney injury, including the acute phase, when hospitalized patients experience critical complications such as sepsis. In vivo studies have shown that treatment with MaR1 can be a therapeutic strategy in this area: in an experimental model of acute septic kidney injury in vivo on male mice, it has been shown that in the presence of a pro-inflammatory stimulation by a single intraperitoneal injection of LPS (10 mg/kg), the administration of MaR1 reduced the levels of TNF-α, IL-6, and IL-1β; downregulated the expression of BAX and caspase-3; and increased the expression of BCL-2 in damaged renal tissue. In addition, the same study highlighted the antioxidant action of MaR1: it improved the activity of superoxide dismutase in renal tissues and inhibited ROS production. In conclusion, MaR1 attenuated LPS-induced renal inflammation and oxidative stress by inhibiting the NOX4/ROS/NF-κB p65 pathway [175]. In the early stages of kidney injury, MaR1 treatment is also able to enhance antioxidant defenses through increased nuclear translocation of Nrf2 and expression of HO-1, SOD, and NQO-1, thereby attenuating oxidative stress [176]. Another study also investigated the protective role of MaR-1 in the pathogenesis of diabetes-associated chronic kidney injury and its clinical relevance, using male C57BL/6 J mice as an in vivo experimental model in which diabetes was induced by a high-fat diet combined with streptozotocin (STZ) treatment. Human renal proximal tubular epithelial cells (HK-2) were then used as an in vitro experimental model, and serum MaR1 levels were finally analyzed in a clinical study involving 104 subjects with type 2 diabetes (T2DM). In these subjects, serum MaR1 levels decreased significantly during the course of kidney disease compared to unaffected subjects. In CKD patients, LGR6 levels were significantly lower compared to healthy controls as in mouse models, but MaR1 treatment restored these levels at both protein and mRNA levels. Furthermore, in high glucose-treated cell models, MaR1 counteracted the reduction of LGR6, suggesting that the protective effects of MaR1 may be linked to the enhancement of LGR6 function. Knocking down LGR6 in cells blocked the beneficial effects of MaR1 on reducing ROS and inflammation, further confirming that LGR6 is essential for the actions of MaR1, reduced hyperglycemia, and slowed the pathological progression of kidney damage. The fact that this study showed that decreased serum MaR1 levels are closely related to the development of CKD in the presence of diabetes suggested that MaR-1 can be considered a predictor and a potential therapeutic target for this disease [177]. One of the key mechanisms through which MaR1 exerts its effects is an antioxidant pathway mediated by cAMP and SOD2. MaR1 treatment increased cAMP and SOD2 levels in both cells and kidneys of mouse models, reducing ROS accumulation and modulating inflammation. These findings support the hypothesis that MaR1 ameliorates DKD through an antioxidant mechanism mediated by the LGR6 receptor, specifically by modulating the cAMP/SOD2 pathway [178].

#### 6.3.2. Protectins

Protectins (PDs) are lipid mediators produced by neutrophils, macrophages, and T cells from docosahexaenoic acid (DHA). They are of great biomedical interest because of their ability to limit the extent and duration of the acute inflammatory response and to regulate tissue regeneration [179]. Among several products derived from DHA metabolism, a lipidomic study in 2002 by Serhan et al. also reported biosynthetic and structural information of a product, later named protectin D1 (PD1) [178]. PD1 is a compound of interest since it has a pro-resolving effect on the inflammatory state and defends the host against bacterial and viral infections [179,180]. The loss of balance between anti-inflammatory and pro-inflammatory factors is often at the basis of the loss of kidney function in CKD, but the experimental evidence highlighted how the administration of PD1 can contribute to the reduction of kidney damage, leading to an anti-inflammatory response that is critical for the resolution of kidney damage [181]. In kidney disease, ischemic-inflammatory damage to the kidney plays an important role in regulating this balance; this has been demonstrated using a mouse model of ischemic kidney injury, in which polymorphonuclear cells produce both bioactive D-series and DHA precursor during the inflammatory response. First, the precursor DHA was shown to be present in the kidney before and after ischaemic damage. However, greater amounts of PDs and resolvins were produced in the post-ischaemic kidney, and the administration of these compounds protected the kidney from further damage by reducing leukocyte influx and the increase in serum creatinine with a reduction in post-ischaemic renal fibrosis [182]. Based on these findings, it is likely that endogenous anti-inflammatory compounds involved in resolving the inflammatory response play a crucial role in the progression of both acute and chronic kidney injury. Notably, after the onset of ischemic injury, higher doses of PD1 are necessary to achieve therapeutic levels in damaged kidneys compared to the doses of RVs required. Further research is needed, as a single dose of 10 μg of PD1 was selected for the post-injury phase of the study [183].

PD1 has also been shown to have renoprotective and antioxidant effects in CKD, in particular, acting on the heme oxygenase-1 (HO-1) pathway, which has been shown to be protective both in rodent and in human kidney disease models [184]. In fact, HO-1 is an enzyme involved in the degradation of toxic heme to bile pigment biliverdin and carbon monoxide (CO), showing antioxidant and anti-inflammatory properties, as well as to admeliorate the circulatory flow in the rat model [164]. Furthermore, a study by Hassan and Gronert on in vitro and in vivo models suggests that PD-1 induces the HO-1 pathway in mesangial cells [185].

However, the administration of PDs, which retain anti-inflammatory and antifibrotic properties, could be therapeutically valuable in treating renal injury. This approach may facilitate the early resolution of damage and help prevent the irreversible progression of lesions in chronic kidney disease (CKD).

#### 6.3.3. D-Series Resolvins

Endothelial cells, located in the inner wall of blood vessels, are a target of RvD1 and RvD2. RvD1 is among the most extensively studied resolvins due to its demonstrated pro-resolving effects in various acute inflammatory diseases in animal models. These conditions include lung injury, kidney damage, pancreatitis, hepatitis, and peritonitis [186]. RvD1 also upregulates the expression of ZO-1, occludin, and narrow-junction proteins to protect against endothelial barrier dysfunction via the IκBα pathway [187]. RvD1 can enhance macrophage efferocytosis of apoptotic neutrophils. The same effect on M2 macrophages is also exerted by RvD3 and RvD5 [188]. RvD1 has also effects on podocytes during nephropathy that is characterized by proteinuria and glomerulosclerosis, induced by adriamycin treatment. This injury results in a progressive loss of synaptopodine, an actin-associated protein that plays a role in the shape and motility of actin-based cells in renal podocytes [189]. Synaptopodine downregulation in course of nephropathy can be modulated by early administration of RvD1. Similarly, in a podocyte cell line, resolvin D1 was able to prevent synaptodin downregulation induced by TNF-α treatment [190]. Supplementation with 4 g/day of n-3 fatty acids for 8 weeks enhances the synthesis of SPMs (specialized pro-resolving mediators) that promote inflammation resolution. Specifically, n-3 fatty acids significantly increase the levels of RvD1 and the upstream precursors of RvE and RvD, 18-HEPE, and 17-HDHA, respectively [191]. RvD1 has been shown to have a protective effect against intestinal damage mediated by certain drugs, such as NSAIDs, as demonstrated by an in vivo study conducted in 2021. Indeed, this study showed that RvD1 derived from 12/15-lipoxygenase contributes to mucoprotection against NSAID-induced small intestinal damage by exerting anti-inflammatory effects through activation of the ALX/FPR2 receptor [192]. Furthermore, exogenous supplementation of RvD1 protected the small intestine from NSAID-induced damage. In contrast, inhibiting 12/15-lipoxygenase, the enzyme responsible for producing resolvin D1 and blocking ALX/FPR2, worsened NSAID-induced small intestinal damage. These findings align with previous studies suggesting that RvD1 protects against NSAID-induced intestinal damage by suppressing the expression of TNF-α and IL-1β through the inhibition of the TLR4-NF-κB and NLRP3 inflammasome signaling pathways [193]. This mechanism on toll-like receptors, which are implicated in the progression of kidney damage, was also highlighted in another study, underlining the important role of RvD1 in counteracting disease progression [194]. RvD2 regulates NO production and adhesion molecule receptor expression in endothelial cells [195].

In a study conducted in 2014 by Katakura M. et al., it was shown that dietary supplementation of a ω-3 PUFA (EPA + DHA) formulation in SHRcp rats (used as an animal model of metabolic syndrome) was able to attenuate renal dysfunction and tissue damage [196]. Supplementation with EPA + DHA would also appear to stimulate the levels of DHA in plasma and its derivatives (PD1, RvD1, and RvD2) in the kidneys in contrast to supplementation with EPA alone. The fact that the increased production of DHA derivatives was associated with a reduction in glomerular damage suggests that these compounds could contribute to the suppression of the progression of renal dysfunction in metabolic syndrome types.

## 7. Conclusions

**Figure 3:**
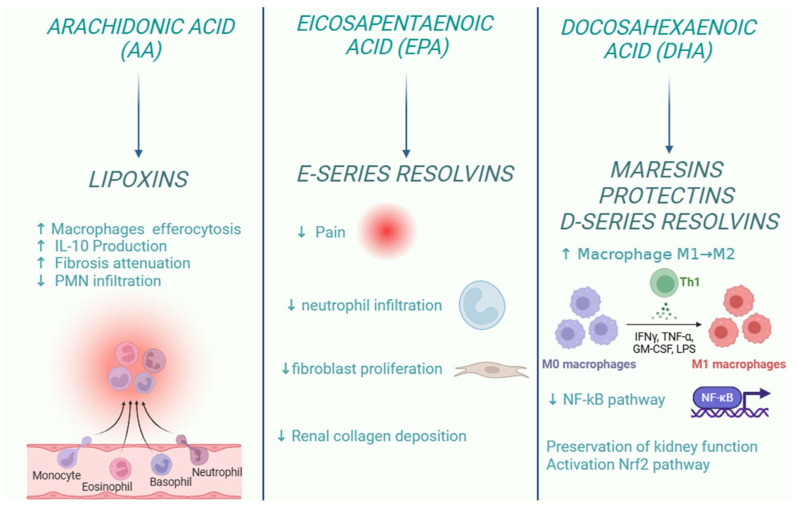
Role of specialized pro-resolving lipid mediators in the resolution of kidney inflammation.

Inflammation is a central element in kidney disease, and its resolution is crucial to repristinate the physiological renal function after damage. In this context, it is important to note that SPMs play an important role in the resolution of the inflammatory response. New therapeutic strategies based on SPM aim to reduce the inflammatory response associated with kidney disease, acting on several fronts: limiting neutrophil recruitment (PMN), relieving pain, modulating fibrosis, stimulating macrophage-mediated efferocytosis, and preserving renal function through the activation of antioxidant pathways (Figure 3).

Supplementation with SPM precursors has attracted increasing interest as a therapeutic approach in acute and chronic inflammatory diseases, as evidence suggests that levels of these mediators are reduced in patients suffering from inflammatory and immune disorders. In particular, in the context of renal disease, experimental evidence suggests that SPMs, both endogenous and synthetic, are effective in preserving renal function (Table 1).

CKD currently represents a serious global health challenge, with a shortage of effective treatments. Chronic dialysis is a key strategy to support many patients, especially in the advanced stages of kidney disease. However, several toxic products are not easily eliminated by this treatment. Moreover, the high costs associated with dialysis highlight the urgency of developing alternative therapies. Renal transplantation remains a solution in the most severe cases, but the increasing shortage of organs and limited access to effective immunosuppressive drugs, which could reduce side effects, emphasise the importance of early diagnosis. However, this therapy is associated with risk factors, such as tumour occurrence and fibrosis, and the long-term effects of these treatments are unknown. Early diagnosis reduces the need for complex treatments and also reduces related complications. Research is exploring new therapeutic strategies, such as the use of embryonic stem cells for renal regeneration or gene therapy. Stem cell therapies, in particular, induced pluripotent stem cells, mesenchymal stem cells, and renal stem cells, could stimulate the regeneration of damaged kidney tissue and new nephrons, thus reducing the need for dialysis and transplantation. However, this therapy is associated with risk factors such as cancer and fibrosis, and the long-term effects of these treatments are unknown. Similarly, new drugs that act on specific genes could enable personalised treatments and improve patients’ quality of life [204]. New therapeutic strategies based also on pro-resolving mediators aim to slow down the complications associated with kidney disease, providing valuable support against this global health challenge. The therapeutic approach that exploits the resolving properties of these mediators has the potential not only to stop the action of pro-inflammatory mediators but also to actively contribute to the resolution of the inflammatory process without suppressing the host’s immune response. Although preclinical studies have led to the development of compounds that are more resistant to metabolic inactivation than their endogenous homologues, further studies are certainly needed to improve the stability of these lipid compounds. Formulations based on liposomes, lipid nanoparticles, or nanotechnologies are currently under study to obtain greater stability, bioavailability of SPMs, and their targeted delivery to inflamed tissues [182]. Such research could shed light on aspects that are still unclear, such as the optimal timing and methods of administration of these mediators in the context of renal disease. Given the multiple actions of SPMs in different organs, including the kidney, the identification of metabolically stable mimetics molecules could be a significant breakthrough, increasing their potential to promote the resolution of inflammation. In this way, defects in the resolution of inflammation, an aspect that characterizes many chronic human diseases, could be addressed. Incorporating these compounds into the treatment of kidney disease could open up new therapeutic perspectives and significantly improve the management of chronic inflammatory diseases.

## Figures and Tables

**Table 1:** Summary of studies based on SPMs in various experimental models of renal disease.

SPM Family	Experimental Model	Effects on CKD
Lipoxins	Renal ischemia and reperfusion injury (mouse)	Reduction of fibrosis [195,197]
Lipoxins	Activation of renal fibroblasts (rat)	Reduction of proliferation [195]
Lipoxins	Obesity-induced glomerulopathy (mouse)	Reduction in albuminuria and renal collagen deposition [198]
Lipoxins	Renal ischemia reperfusion injury (rat)	Activation of multiple antioxidant pathways (es. HO-1) [198]
Maresins	Renal ischemia (mouse)	Inhibition of NF-kB activity [174]
Maresins	Reperfusion Injury (mouse)	Protection of renal function [174]
Protectins	Renal ischemia and reperfusion injury (mouse)	Protectins, especially PD1, mitigate kidney injury [182]
Protectins	Injured kidney (mouse)	Reduction of PMN infiltration [182]
Resolvins	Renal ischemia reperfusion injury (mouse)	Attenuation of the renal injury and decreased leukocyte infiltration by RvD1 [199]
Resolvins	Unilateral ureteral obstruction (mouse)	Attenuation of fibroblast proliferation, collagen deposition, and fibrosis formation by RvE1/D1 [200]
Resolvins	Acute renal injury (mice)	Reduction of pro-inflammatory mediator (LPS) by RvD2 [201]
Resolvins	Acute renal injury (mice)	Activation of Nrf2-mediated antioxidant pathways by AT-RvD1 administration [202]
Resolvins	Acute renal injury (mice)	Restoration of renal tubule function, inhibition of NF-kB release, and IL-6 activation by RvD1 [188]
Resolvins	Podocyte injury (mouse)	RvD1 reduces podocyte-NLRP3 inflammasome activation and enhances podocin expression [203]
Resolvins	Adriamycin-mediated nephropathy (mice)	RvD1 protects podocytes via 14-3-3beta acetylation [188]
Resolvins	Myocardial infarction-induced cardiorenal disease (mice)	Attenuation of MI-induced inflammation and improvement in the podocyte nephrin expression by RvD1 [160]
